# De novo assembly of *Dekkera bruxellensis*: a multi technology approach using short and long-read sequencing and optical mapping

**DOI:** 10.1186/s13742-015-0094-1

**Published:** 2015-11-26

**Authors:** Remi-Andre Olsen, Ignas Bunikis, Ievgeniia Tiukova, Kicki Holmberg, Britta Lötstedt, Olga Vinnere Pettersson, Volkmar Passoth, Max Käller, Francesco Vezzi

**Affiliations:** 1 Department of Biochemistry and Biophysics, Science for Life Laboratory, Stockholm University, Box 1031, 171 21 Solna, Sweden; 2 Uppsala Genome Center, NGI/SciLifeLab, Department of Immunology, Genetics and Pathology, Uppsala University, BMC, Box 815, SE-752 37 Uppsala, Sweden; 3 Department of Microbiology, Swedish University of Agricultural Sciences, Box 7025, SE-75007 Uppsala, Sweden

## Abstract

**Background:**

It remains a challenge to perform de novo assembly using next-generation sequencing (NGS). Despite the availability of multiple sequencing technologies and tools (e.g., assemblers) it is still difficult to assemble new genomes at chromosome resolution (i.e., one sequence per chromosome). Obtaining high quality draft assemblies is extremely important in the case of yeast genomes to better characterise major events in their evolutionary history. The aim of this work is two-fold: on the one hand we want to show how combining different and somewhat complementary technologies is key to improving assembly quality and correctness, and on the other hand we present a de novo assembly pipeline we believe to be beneficial to core facility bioinformaticians. To demonstrate both the effectiveness of combining technologies and the simplicity of the pipeline, here we present the results obtained using the *Dekkera bruxellensis* genome.

**Methods:**

In this work we used short-read Illumina data and long-read PacBio data combined with the extreme long-range information from OpGen optical maps in the task of de novo genome assembly and finishing. Moreover, we developed NouGAT, a semi-automated pipeline for read-preprocessing, de novo assembly and assembly evaluation, which was instrumental for this work.

**Results:**

We obtained a high quality draft assembly of a yeast genome, resolved on a chromosomal level. Furthermore, this assembly was corrected for mis-assembly errors as demonstrated by resolving a large collapsed repeat and by receiving higher scores by assembly evaluation tools. With the inclusion of PacBio data we were able to fill about 5 % of the optical mapped genome not covered by the Illumina data.

**Electronic supplementary material:**

The online version of this article (doi:10.1186/s13742-015-0094-1) contains supplementary material, which is available to authorized users.

## Background

In the last decade we have witnessed an unprecedented development in sequencing technologies. This is sometimes referred to as the next-generation sequencing (NGS) revolution. Year by year, new technologies and chemistries have, to varying degrees, enabled increased throughput, read lengths and sequence quality. Currently there is a wide range of technologies and companies that allow sequencing and genomics analysis at a speed and with a throughput thought impossible only few years ago. In this work we focus our attention on three established technologies and their compatible tools: Illumina [1], PacBio [2] and OpGen [3]. However, the methods presented here can easily be extended and applied to similar and/or emerging technologies, e.g., IonTorrent [4], Oxford Nanopore [5] and BioNano [6]. The Illumina sequencing technology has become a leading tool in a wide range of application areas. Among others, Illumina is used for whole genome resequencing, haplotype phasing and identification of structural variations. Illumina technology is also widely used in de novo genome assembly projects. Despite the short read length, Illumina is used to quickly and cheaply obtain high genome coverages [7].

In 2011 Pacific Biosciences released the first commercially available long-read sequencer based on single-molecule real-time (SMRT) sequencing technology. In contrast to the short (i.e., 150 to 300 bp) Illumina reads, the PacBio RS II instrument produces average read lengths ranging from 10–15 kb, with the ultra-long reads exceeding 50 kb. Such unprecedented read lengths are ideal for de novo assembly. However, long reads are also a key in studying structural variations or investigating isoforms by sequencing full-length intact transcripts [8–11].

Another technology is optical mapping [12–16], a method based on mapping the position of enzyme restriction sites along the sequence of the genome as observed by fluorescence microscopy, which was automated [17, 18] to achieve high-throughput solutions amenable to the analysis of complex genomes. This technique allows the production of extremely long (hundreds of kbp) restriction maps, which to date has been applied to, e.g., alignment to a reference sequence to identify structural variations in bacteria [19–22] and in humans [23–25]. In the field of de novo assembly, whole genome mapping has been used for scaffolding prokaryote assemblies [26, 27] and a fungus assembly [28], but also assemblies of complex eukaryotes such as the domestic goat [29] and maize [30]. Optical mapping was also used for refining the mouse reference genome [31].

Yeasts are unicellular fungi, with a high diversity and a high phylogenetic distance. They are essential for a number of biotechnological applications, for the functioning of natural ecosystems or can act as human and animal pathogens [32, 33]. Since they have relatively small and compact genomes they are also ideal model organisms to study eukaryotic genome evolution. Indeed, the first sequenced eukaryotic organism was the yeast *Saccharomyces cerevisiae* in 1996 [34]. In 2010, about 40 yeast species had been sequenced and reported [32]. After the establishment of NGS, the number of sequenced yeast genomes rapidly increased, and today, for some yeast species, the intraspecific genome diversity between strains can be determined [33, 35–37]. However, short-read draft assemblies are often comprised of hundreds of unsorted and disordered contigs. This makes it very difficult, or impossible, to investigate chromosome rearrangements such as inversions, duplications or chromosomal translocations, which play an important role in fungal evolution [32]. Moreover, pulsed field gel electrophoresis studies have shown considerable chromosome polymorphisms among strains of fungal species [38–41], thus making de novo assembly a much more difficult and complex task.

In this work we will show how combining multiple technologies in a de novo sequencing project – in effect exploiting their individual strengths – is an optimal strategy to improve the quality of the resulting assembly. In doing so we will perform an extensive validation of obtained assemblies. As a by product, we also present a semi-automated de novo assembly pipeline, dubbed ‘NouGAT’, which was instrumental to this work. This pipeline is currently in use at the National Genomic Infrastructure hosted at SciLifeLab in Stockholm to assemble hundreds of genomes every year. As a proof-of-concept, we applied our approach to carry out a de novo assembly of the yeast genome, *Dekkera bruxellensis*.

## Methods

### De novo assembly pipeline

**Fig. 1:**
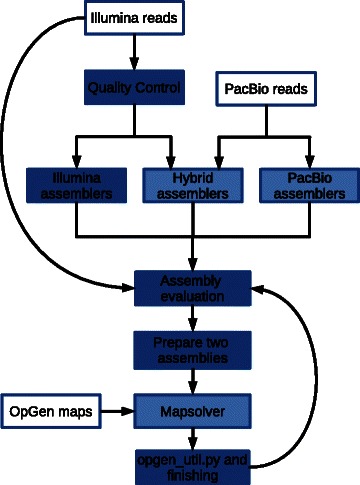
Bioinformatic workflow overview. There were three entry points of data, shown by boxes with white shading: Illumina read data, PacBio read data and OpGen optical map data. Boxes shaded in dark blue show work done by the assembly pipeline in a semi-automated fashion: quality control (and trimming) of short-read Illumina data, Illumina-only assemblers, evaluation of assembly quality (for all assemblies) using feature-response curves and standard metrics, preparing two chosen assemblies for in silico digestion and optical map placement and finally using open_util.py to generate an assembly from the scaffold-optical map placement coordinates. Work done outside the assembly pipeline is shown as boxes with light blue shading: the PacBio-only assemblies, the PacBio-Illumina hybrid assemblies and the operation of OpGen’s Mapsolver software for in silico digestion and placement of scaffolds and their placement on optical maps

We describe a semi-automated de novo assembly pipeline dubbed NouGAT [42], developed at the National Genomics Infrastructure (NGI) at SciLifeLab in Sweden. The aim of this tool is to easily (i) pre-process the sequencing data, (ii) assemble input data in a semi-automated way, (iii) evaluate and rank assemblies, and (iv) use information from optical maps to improve the quality of the draft assembly. NouGAT’s design is based on the findings of the Assemblathon 1 and 2 challenges [43, 44], and by the evaluation study GAGE [45]. Below we demonstrate NouGAT, by assembling the genome of *D. bruxellensis*, (see Fig. 1).

Pre-processing of reads is of great importance for assembly quality, as previously demonstrated by the GAGE study [45]. It is also essential to assess the quality of the reads to spot problems in the steps prior to assembly, e.g*.,* DNA extraction, library construction and sequencing. For short-read data, the pipeline uses Trimmomatic [46] for removing adapter contamination and low quality regions. This has been shown to prevent the generation of adapter-chimeric contigs and to increase assembly contiguity [46]. Using the trimmed reads as input, the pipeline plots the k-mer abundance as generated by ABySS [47] and the quality metrics generated by FastQC [48].

The assembly sub-pipeline is created to enable a variety of assembly programs to be run. As previously shown in the Assemblathon and GAGE studies, different assemblers can result in completely different assembly qualities. However, the large number of assemblers, and the large number of user-definable parameters, can make this a difficult task. NouGAT allows the user to run a number of assemblers (seven are currently supported) by specifying a single configuration file. Currently only Illumina-only assemblers are supported, but ongoing work is in progress to extend this subpipeline to PacBio and hybrid assemblers.

For evaluating assemblies, the standard contiguity and size metrics (e.g*.,* N50, average contig size, etc.) may give a false representation of its correctness [49]. As an example, an assembly composed of few but very long contigs (i.e., a highly connected assembly) might not always be the best representation of the underlying genome [45] because longer contigs could be the results of a too-eager assembly strategy. A handful of tools exists to gauge assembly quality and correctness on the basis of more reliable metrics [49–51]. The majority of these tools try to reconstruct the read layout and to identify areas of the assembly that are likely to contain mis-assemblies. We decided to employ feature-response curves (FRC) [49]. FRC uses anomalously mapped paired-end and mate-pair reads to identify suspicious areas, called features. Subsequently, features are tallied for each contig, along with the estimated contig genomic coverages. These points are ordered by decreasing contig size and plotted by accumulating the number of features. The resulting plot is, in some aspects, similar to a receiver operating characteristic (ROC), where the assembly with the steepest curve is likely to contain fewer mis-assemblies.

The strategy for assembly refinement using optical maps is to select the two best performing assemblies and place their contigs on the optical maps. This entails manual curation using OpGen’s Mapsolver software. Subsequently, a consensus sequence is generated for each assembled optical map using a utility script found in NouGAT.

## Results

### De novo assembly

A total of seven assemblies were generated using Illumina and PacBio sequencing data (for a summary of computational resources used see Additional file 1: Table S4). We used these two data sets both in isolation and combined. To generate assemblies from only Illumina reads, we used ALLPATHS-LG [52], ABySS [47], and SOAPdenovo [53]. For assembly of PacBio reads only, HGAP [8] and FALCON [54] were used. Illumina-PacBio hybrid assemblies were generated by AHA [55] and CABOG (using pacBioToCA error correction by Illumina reads) [56]. For assemblers using a De Bruijn Graph method with a mandatory k-mer size parameter (ABySS and SOAPdenovo), we tested a range of k when running SOAPdenovo, and found k = 61 to be optimal (see Additional file 1).

We computed standard contiguity metrics (Table 1) for all assemblies. Table 1 shows that ALLPATHS-LG gave the most well connected Illumina assembly, i.e., greater N50 and fewer but longer contigs. In comparison, the ABySS assembly had the lowest N50 number and more numerous but shorter contigs. In terms of N50, the SOAPdenovo assembly can be regarded as being better connected than the ABySS assembly; however, a large majority of the assembly consists of contigs less than 1 kbp in length. When considering PacBio only assemblies, the most connected assembly is the one produced by HGAP, which has an N50 four times shorter than that produced by ALLPATHS-LG. FALCON performed noticeably worse than HGAP, with a much lower assembly length (see Table 1) and a lower N50. However, FALCON is experimental and might not be suitable for the input data, and/or it was used with non-optimal parameters. AHA fared best among the hybrid-assemblies.

**Table 1:** Standard contiguity metrics

Name	#scaff	#scaff > 1000	N50	max_scf	asm_lgth	asm_lgth >1000
Chr1-7	15	15	3706655	4993496	17319971	17319971
Chr1-4	12	12	3706655	4993496	14763326	14763326
soapdenovo	66606	396	263103	1117059	25625475	13038098
allpaths	352	349	610180	1845683	13885397	13882423
abyss	6523	1061	46286	581122	18806852	18097355
HGAP	308	308	147223	776319	14719721	14719721
FALCON	410	405	30567	152911	10731982	10728849
AHA	241	241	201733	758433	15105135	15105135
pacBioToCA	1579	1579	157083	692841	17014896	17014896

Columns from left to right: name of the assembly, number of scaffolds, number of scaffolds after removing those under 1 kbp, N50, N80, the longest scaffold, assembly length, assembly length after removing scaffolds under 1 kbp

**Fig. 2:**
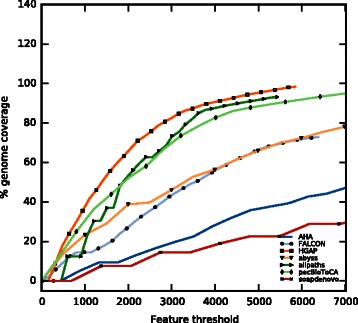
Feature response curves. Feature response curves (FRC) for assemblies considered for optical map placement. On the x-axis is the total number of features normalised for the assembly contig count. On the y-axis is the coverage based on the estimated genome size of 14,719,721 bp (size of the first completed assembly, HGAP)

**Fig. 3:**
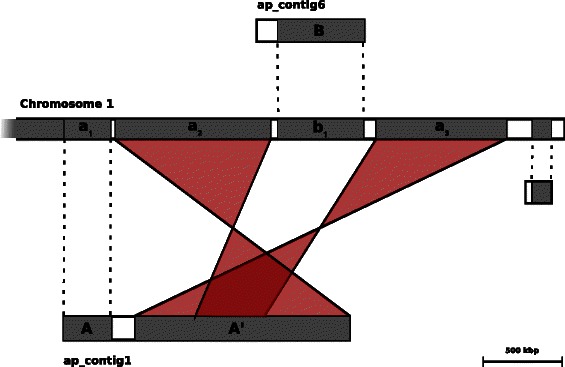
Placement of ap_contig1 to optical map Chromosome 1. An illustration re-drawn from the output of the OpGen’s Mapsolver software, where in silico digested allpaths-lg contigs are placed to the optical map Chromosome 1. It shows a complex rearrangement where flaws in the allpaths-lg assembly are corrected. The 1.38 Mbp region *A´* of ap_contig1 is a collapsed repeat structure, which the optical map was able to resolve and subsequently could be placed to regions *a*_*1*_ and *a*_*2*_ of Chromosome 1. This map placement is highlighted in transparent red for clarity and shows that the sequences were placed in inversed orientation. Furthermore, *a*_*2*_ and *a*_*3*_ are flanking the placed sequence *b*_*1*_, originating from the *B* region of the contig ap_contig6. On the left flank of *B* is an unplaced region whose restriction enzyme cuts could not be aligned to the cuts made by the Argus system, and is likely the result of mis-assembly

In the absence of a reference sequence, it is difficult, if not impossible, to determine the assembly that is most representative for the underlying genome based on the standard contiguity metrics alone. We ran CEGMA on all assemblies to evaluate their gene space (see Fig. 5 and section below for more details). However, CEGMA only helped us to identify SOAPdenovo, FALCON, and AHA as outliers. The remaining five assemblies contained a similar number of core genes. We decided to use FRC analysis to evaluate our assemblies, used in a similar way to that used for the Norway spruce genome [7] and GAM-NGS studies [57]. The cumulative feature curves (Fig. 2) confirmed the poor performance of the less connected assemblies produced by ABySS and FALCON. FRC did, however, overturn the contiguity metrics for most connected assemblies: ALLPATHS-LG and HGAP. FRC also reshaped the order of PacBio assemblers pacBioToCa and HGAP. ALLPATHS-LG was not only the best Illumina assembler, but also generated the assembly with fewest features, i.e., areas of suspected mis-assembly. However, Fig. 2 shows that HGAP was able to cover more of the genome while introducing fewer features. Clearly, the long ALLPATHS-LG contigs accumulate more features than the shorter HGAP contigs, e.g., with 2000 features we were able to cover more than 60 % of HGAP assembly but ‘only’ 50 % of that assembled by ALLPATH-LG. This might suggest that the long ALLPATH-LG contigs are the result of a too-eager assembly strategy (see Fig. 3 and Additional file 1: Figure S2). Remarkably AHA, one of the better connected assemblies, performed much worse than pacBioToCA because of it had a high number of compressed repeat features (Additional file 1: Figure S3).

After a careful analysis of contiguity metrics, CEGMA hits, FRC curves, and coverage plots (Additional file 1: Figure S4 and S5) automatically produced by the NouGAT, we deemed ALLPATHS-LG and HGAP to have produced the best assemblies. Consequently, we chose them for optical map placement.

### Optical map placement

From the OpGen imaging and data processing steps, seven optical maps were obtained, named Chromosome 1, Chromosome 2, etc., spanning about 16.79 Mbp in total. This is an impressive result compared with the 308 and 351 unordered contigs generated by HGAP and ALLPATHS-LG, and with this critical information we were able to both spatially resolve the *D. bruxellensis* genome and to error correct de novo assembled contigs. Using OpGen’s MapSolver software to digest in silico assembled sequences and placement on optical maps, we devised the following strategy: first cover the maps using ALLPATHS-LG contigs of minimum 40 kbp length (shorter fragments cannot be placed as they do not have enough in silico restriction enzyme cuts), and then fill in any remaining gaps using HGAP assembled contigs. Using this method we were able to cover 87 % with contigs, with the remaining unplaced ALLPATHS-LG contigs included as ‘unknown’ sequences.

An interesting feature of note is represented in Fig. 3. In this figure we can clearly appreciate the potentiality of optical mapping when it comes to finishing and error correcting draft assemblies. Chromosome 1 has been assembled to a single restriction map using optical mapping. The figure represents a complex repeat structure, shown schematically as three sequences labelled a_1_, a_2_, b_1_, and a_3_, with a_2_ and a_3_ containing an identical repeat the size of approximately 434 kbp. Thanks to the longer fragment lengths utilised by this method, a complex repeat structure has been resolved (contained in regions a_2_ and a_3_). Neither ALLPATHS-LG nor HGAP (i.e*.,* neither Illumina nor PacBio) alone has been able to correctly reconstruct such a complex scenario. HGAP resulted in 13 small contigs partially covering regions *a*_*2*_ and *a*_*3*_, one of which is placed in both (see Additional file 1: Figure S2). ALLPATHS-LG has been able to produce an extremely long contig, likely using the information inferred from the longest mate-pair library. However, Fig. 3 clearly demonstrates that the long contig, ap_contig1, is the result of wrong decisions made during scaffolding; not only that a complex repeat is collapsed to a single copy, but a 545 kbp region is absent and placed in a different contig (region *B* of ap_contig6). This scenario clearly shows the additional value added by optical maps and the importance of being mindful when presented with long contigs generated from relatively short DNA fragments.

To represent the haploid genome (in the style of a reference genome), we had concerns about the maps for Chromosomes 7, 6 and later 5, since all the ALLPATHS-LG contigs placed therein were duplicates of ones found in the first four maps. The maps for chr5–7 were considerably smaller in size than those preceeding. Furthermore, Mapsolver showed large map-to-map alignments between these two groups (chr1–4 to chr5–7), which strongly suggests that these regions are recombinations.

**Fig. 4:**
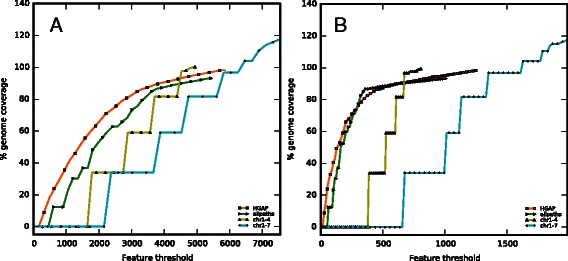
Total and low coverage feature response curves. The total feature response curves (**a**) only shown for HGAP, allpaths, chr1–7 and chr1–4. The decreased number of features when removing Chromosomes 7, 6 and 5 is mostly attributed to regions of low read coverage (**b**)

To test how well chr5–7 are supported by the sequencing data we generated two map-placed consensus sequences: one consisting of sequences for chr1–7 and another of sequences chr1–4. These were processed by the assembly evaluation pipeline, and the feature response curves (Fig. 4) clearly indicated that the assembly for chr1–4 is the best performing assembly, which it owes mainly to the reduction of low coverage regions when the Illumina reads are mapped. It also becomes obvious that chr1–4 is able to cover more of the genome than HGAP (the best performing assembly), while introducing fewer features: approximately 4900 in chr1–4 compared with 5800 in HGAP.

### Validation using CEGMA

**Fig. 5:**
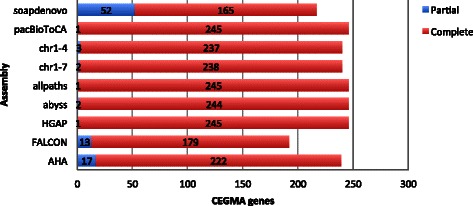
Reported CEGMA gene hits. Barchart showing the number of hits to a set of 248 extremely conserved eukaryotic genes, as reported by CEGMA. Classified as either ‘complete’ or ‘partial’, depending on the alignment percentage

As an extra validation step we ran CEGMA [58], which maps the assembled sequences to a set of 458 highly conserved eukaryotic genes. For the 248 most extremely conserved genes, alignments to the queried assembly are classified as ‘complete’ or ‘partial’ depending on a fixed alignment length threshold. Of the total number of CEGMA hits, allpaths and HGAP performed equally with 246 hits of which one is a partial hit. While the results from CEGMA were not, in our case, essential to the evaluation of the assemblies (over 95 % completion for most assemblies, Additional file 1: Table S1), two observations are remarkable. First, FALCON and abyss, which we earlier established as ‘poor’, are reflected in these results by having a lower completion rate. Second, the final *Dekkera* assembly (chr1–4) received a total of 240 hits, of which three are partial hits (Fig. 5) retaining most of the core genes in an ordered and oriented manner. Further evidence of chr5–7 being artifacts of mis-assembly is the fact that excluding these did not reduce the total number of hits, only a partial loss of one hit. This can also be seen by the higher percentage of orthologous hits in chr5–7 (Additional file 1: Table S1).

### Genome completion using PacBio

We carefully investigated the proportion of optical maps that is assembled exclusively by HGAP. In other words, we wanted to check what we gain by combining Illumina and PacBio assemblies. HGAP contigs were able to add 487 kbp of new sequences, which ALLPATHS-LG was not able to reconstruct. Moreover, 363 kbp out of 532 kbp of ambiguous sequences (gaps and ambiguous base calls) could be replaced using the sequencing information from HGAP contigs. In total, the PacBio data allowed us to resolve slightly more than 5 % (Additional file 1: Table S3) of additional genomic content. We believe that, when automated, this presents an effective strategy for genome finishing.

## Discussion

During a de novo project several decisions need to be taken, often based on little tangible information: which sequencing technology to use, which type of libraries to prepare, what sequencing depth to aim for, which assembler to employ, etc. A poor initial choice can lead to extremely poor results, and these choices are often guided by budget, available technology and/or in-house expertise. The multitude of different tools and approaches to de novo assembly can often lead to an inefficient trial-and-error approach to find acceptable results, prolonging the project and increasing the cost.

This study addressed the problem of the scarcity of methods for efficient scaffolding of genomic contigs into chromosomal units. Rapid development of sequencing technologies exceeded the establishment of pipelines for high-quality draft genome assembly and resulted in fast generation of low-quality genome drafts in public databases [59, 60]. Our study presents a solution to this problem. Using an efficient scaffolding approach guided by application of OpGen optical map placement allowed us to reconstruct the chromosomal makeup of a yeast species. Previously, presentation of a genome on the chromosome level was done only for a limited number of yeast species, and by using expensive and time consuming Sanger sequencing [34]. Other promising alternative scaffolding methods based on the conformation capture (3C) principle were shown to be efficient for several genomes, including that of the yeast *Saccharomyces cerevisiae* [61]. Our approach presents a simplified automated procedure of rapid ordering of PacBio and Illumina-derived contigs according to restriction maps from single microbial DNA molecules. The technique described in this paper can easily be extended to complex eukaryotic organisms. However, it must be taken into account that for larger eukaryotic genomes the steps involved in optical map scaffolding might be laborious and time consuming. Nevertheless, recent publications have shown how optical maps can greatly improve assembly results [29].

In this paper we have demonstrated a method requiring little effort to generate a high-quality draft assembly that can open up new opportunities for assembling complex genomes. In particular, we showed how combining several technologies and using a semi-automated pipeline can easily allow the production of an almost-finished yeast genome assembly. Thanks to their compact genomes and distinct physiological properties, yeasts are ideal model organisms to study evolution [62]. Evolution of a central core of about 4000 genes in the yeast genome has resulted in the origin of various yeast species [63]. Evolutionary events, such as gains and losses of genes were shown to be influenced by their location on a chromosome [64]. Thus, the representation of yeast genomes on the chromosomal scale will allow evolutionary events to be traced and a better understanding of the mechanistic basis behind the versatile diversity of yeast species. While our approach can easily be applied to a wider set of organisms, we assert that it has the potential to bring yeast comparative genomics up from the sequence level [65–68] to the level of chromosomal site analysis. This gives us a tool to extend our understanding of poorly investigated yeast genome structure and function.

The method presented in this study resulted in the determination of a haplotype number of chromosomes in this yeast strain. Analysis of the level of heterozygosity allows us to conclude that the examined genome is more than haploid. One limitation of the presented method is associated with its inability to identify exact ploidy. Additional biochemical methods may resolve ploidy characteristics, such as determining the amount of DNA per cell and its correlation to the genome size.

## Conclusions

In this study we have demonstrated a novel way to combine three high-throughput technologies to produce a high quality assembly of the *Dekkera bruxellensis* genome. We employed an extensive number of assemblies using Illumina, PacBio, and a combination of the two technologies. We did this using a semi-automated pipeline that not only reduced the amount of time needed (in particular bioinformatic operator time) but also made our results easy to reproduce and validate. We used optical maps to resolve the genome on a chromosomal level and to error correct the inherent weaknesses of short-read assemblies, while using a long-read assembly to fill in uncovered regions. A set of utility scripts to produce a chromosome level assembly from optical map placement has been designed and is available together with the semi-automated de novo pipeline. Our de novo pipeline is currently used to process all de novo assembly projects currently sequenced at NGI-Stockholm. Hundreds of genomes per year are assembled, evaluated, and subsequently delivered to our users.

## Availability and requirements

Project name: NouGAT

Project home page: https://github.com/SciLifeLab/NouGAT/

Operating system(s): Platform independent, Linux (64-bit) recommended

Programming language: Python 2.7

Other requirements: Anaconda (https://www.continuum.io/)

License: MIT

Any restrictions to use by non-academics: None

## Availability of supporting data

The sequence data is available in the EBI ENA repository, under the study ERP012947. The data set supporting the results of this article is available in the GigaScience Database [69].

## Additional file

Additional file 1: **Additional figures and tables, including method descriptions.** (PDF 721 kb)

## Abbreviations

ABySS: Assembly By Short Sequences, assembly software
bp: Base pair
CEGMA: Core Eukaryotic Genes Mapping Approach, assembly evaluation software
chr: Chromosome
FRC: Feature response curves
HGAP: Hierarchical Genome Assembly Process, assembly software
N50: The length of the shortest contig greater than or equal to 50 % of the genome length
NGI: National Genomics Infrastructure
NGS: Next-generation sequencing
