# Supplementary material for: De novo assembly of Dekkera bruxellensis: a multi technology approach using short and long-read sequencing and optical mapping
Source: Gigascience. 2015 Nov 26;4:56. doi: 10.1186/s13742-015-0094-1 (PMC4661999; doi:10.1186/s13742-015-0094-1)
Supplement: Additional file 1: — Additional figures and tables, including method descriptions. (PDF 721 kb) [file 13742_2015_94_MOESM1_ESM.pdf]

Methods

Miscellaneous

Strains and culture media

We used the industrial isolate of *D. bruxellensis* CBS 11270 (Blomqvist et al. 2010). Yeast extract–peptone–dextrose medium (YPD) was prepared as described earlier (Tiukova, Eberhard, and Passoth).

Cultivation

D. bruxellensis cultures were incubated in 50 ml YPD in 200 ml shake-flasks with shaking (200 rpm) at 30°C for approximately 20 hours.

K-mer length selection

To assess what effect differing k-mer lengths have on assembling our data, we produced ten SOAPdenovo assemblies using a variety of k-mer input parameters: from k=43 to k=97, in increments of six. FRC curves (Additional Figure 6) plotted for these, we see that the quality is increasing with higher k values but reaches a plateau at k=61. The standard contiguity metrics show that k=61 to be superior (Additional Table 2).

DNA sequencing

A summary of all the sequenced libraries can be found in Additional Table 5.

DNA extraction

One millilitre of *D. bruxellensis* culture grown in 50 ml YPD at 30°C to OD 10 was harvested. Cell pellet was mixed with 100 µl protoplasting buffer (100 mM Tris-Cl (pH 7.5), 10 mM EDTA, 0.2 µl beta-mercaptoethanol, 10 units of lyticase) and incubated at 37°C for 3 hours. One hundred microlitres of lysis buffer (200 mM NaOH, 1 % SDS) was added to cell suspension and incubated at 65°C for 20 minutes. After being rapidly cooled on ice, suspension was mixed with 100 µl of 5 M potassium acetate buffer (pH 5.4) and centrifuged. Supernatant was harvested and mixed with 200 µl ice-cold 2-propanol and incubated on ice for 30 min and centrifuged. DNA pellet was washed with 300 µl of 70 % ethanol and dried. DNA was dissolved in water.

Short-read sequencing

Four libraries were prepared for Illumina Hiseq 2500 sequencing: two paired-end libraries using TrueSeq DNA kit (Illumina, CA, USA) with 150bp and 650bp insert sizes, two mate-pair libraries using Nextera mate pair kit (Illumina, CA, USA) with 3kbp insert size and one library with 5kbp insert size.

Preparation of the paired-end libraries was made according to the manufacturers specifications, with the following changes: protocols were automated using an Agilent NGS workstation (Agilent, CA, USA), all the purification and gel-cut steps were replaced by a magnetic bead clean-up method (Lundin et al. 2010; Borgström, Lundin, and Lundeberg 2011) and fragmentation was performed using a Covaris S2 instrument (Covaris Inc., MA, USA).

The mate-pair libraries were constructed using using the Gel-Plus protocol following the instructions given by the manufacturer. Tagmentation cleanup was performed using Genomic DNA Clean & Concentrator (Zymo Research, CA, USA). Size selection was made between 2 and 5 kbp, and between 4 and 7 kbp on a Blue Pippin (Sage Science Inc., MA, USA) using the 0.75% 1-10 kb Gel Cassette with Marker S1 (2-6kb and

3-10kb). Final library clean up was automated and performed on a MBS 1200 pipetting station (Nordiag AB, Sweden) using magnetic bead clean-up methods (Lundin et al. 2010; Borgström, Lundin, and Lundeberg 2011). The short mate-pairs were amplified in 10 to 13 PCR cycles, and the long mate-pairs were amplified in 14 cycles.

Long-read sequencing

Genomic *D. bruxellensis* DNA was used to create two libraries of different fragment length. DNA for the 2kb library was fragmented by sonication using the Covaris S2 system and the DNA for 10kb library was fragmented using a HydroShear® DNA Shearing Device (GeneMachines). Fragmented DNA was end-repaired and adaptors were ligated to generate SMRTbells™ for circular consensus sequencing. Libraries were exo-treated for product clean up following 2 kb and 10 kb template preparation protocols provided by the manufacture. Sequencing primer and P4 polymerase were annealed to the SMRTbell™ libraries and bound to magnetic beads. The libraries were loaded on 2 SMRTcells™ per fraction, using magbead loading and sequenced on the PacBio RS II system using C2 chemistry and a 120 minutes movie time.

Optical mapping

Sample preparation

Yeast chromosomes for analysis in OpGen's Argus system were prepared by a modification of the chromosome embedment in agarose plugs procedure by Carle and Olson (Schwartz and Cantor 1984; Carle and Olson 1985). Cells were grown in 50 ml YPD at 30°C to an optical density at 600 nm (OD_{600}) of 10, harvested and washed in 5 ml of water, 7 ml of 10 mM EDTA and finally in 7 ml of Sortrisca solution (0.1 M Tris-Cl pH 7.5; 10 mM $CaCl_2$; 1.2 M sorbitol). The pellet was incubated for 30 minutes at 30°C in a solution prepared by mixing of 10 ml of Sortrisca solution, 2 mg of lyticase and 20 µl of mercapto-ethanol. The cells were washed twice in 7 ml Sortrisca solution. $5*10^9$ cells resuspended in 1 ml Sortrisca were mixed with 2 ml of agarose solution, cooled to 45°C, prepared by mixing of 20 mg low melting point agarose in 0.125 M EDTA. Cells and agarose mixture was poured into the 10-Well Disposable Plug Mold (Bio-RAD, N170-3713). After solidification the plugs were incubated for 30 min at 4°C, removed from form and transferred into NDS-buffer (0.5 M EDTA, 0.1 M Tris, 1% Na lauroyl sarcosine). The plugs were incubated for two hours with buffer change every 30 minutes. Subsequently, the plugs were incubated in a series of buffers: proteinase solution (1 mg Proteinase K in 3 ml of NDS buffer) at 50°C for 24 hours; LET-buffer pH 7.5 (0.5 M EDTA, 0.1 M Tris) for 5 minutes and Rnase solution (1250 U of T1-Rnase in 3ml LET-buffer) at 37°C for 24 hours. Prepared plugs were stored at 4°C.

Data generation

The Argus system (OpGen®) was used to obtain seven optical maps, using the restriction enzyme KpnI and four HD MapCards. This system fully integrate wet lab chemistry with automatic collection of fluorescence microscope images. To better facilitate the manual editing of the contig-optical map placements in OpGen's MapSolver™ software, the two assemblies selected for this process were trimmed to a minimum contig size of 40 kbp. The output from MapSolver is a report containing the placement coordinates of the de novo assembled contigs relative the optical map, along with their relative orientation. From this, we used the 'opgen_util.py' script of de novo scilife package. It produces a single continous sequence for each optical map by simply translating the placed contig coordinates to the map coordinates and performing inversions where reported. Sequence overlaps between the two placed assembly contigs, in part reduced by the manual editing, was resolved by always selecting the sequences with the fewest ambiguous basecalls.

Program versions and commands

Versions

- trimmomatic/0.30
- fastqc/0.10.1
- abyss/1.3.5
- allpathslg/49618
- SOAPdenovo/2.04-r240
- FRC_align/4bfa2f8 ^
- qaCompute/95c8fd7 ^
- cabog/8.1
- OpGen(r) MapSolver/v.3.2.4
- FALCON/5c46b5f ^
- cegma/2.4.010312
- BWA/0.7.4
- samtools/0.1.19
- picard/1.92

^ Git commit hash

Commands

See github repository: `https://github.com/remiolsen/vp2015`.

Additional illustrations

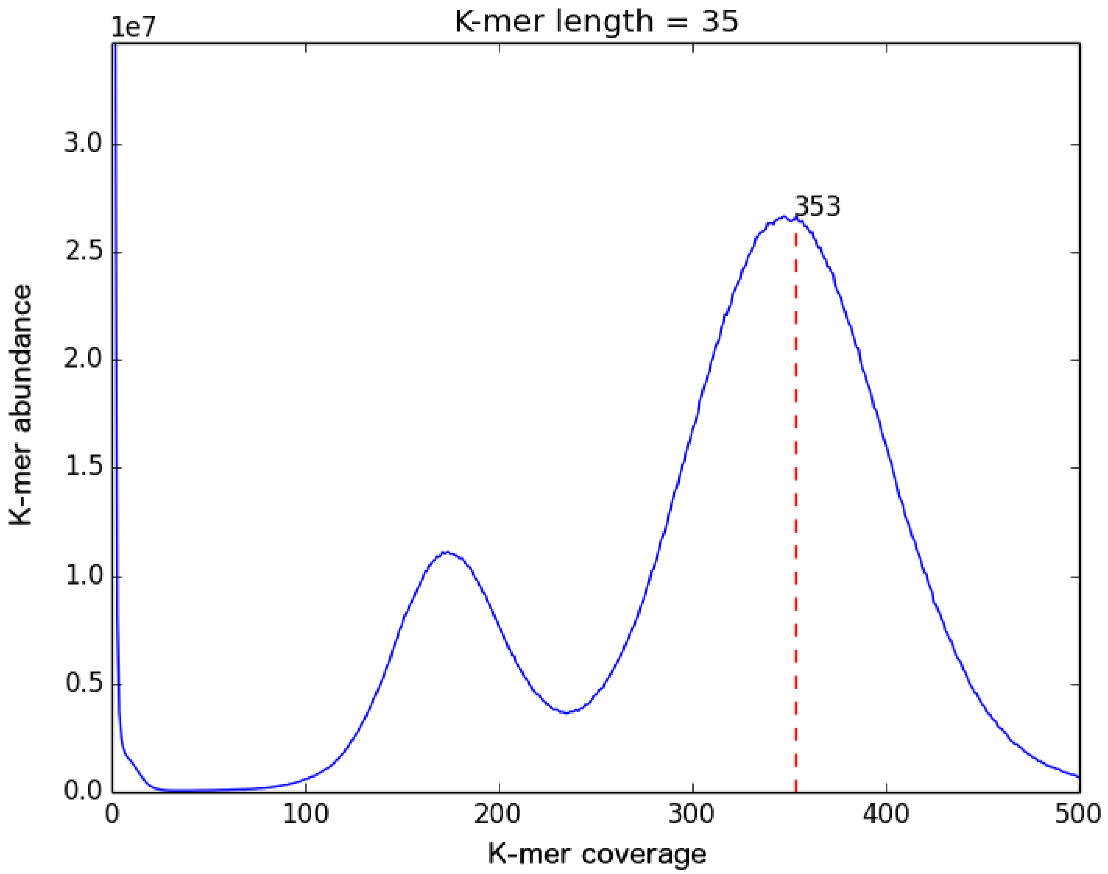

Figure 1. K-mer abundance plot for the PE 150bp insert library, for k = 35. A clear and bell shaped peak can be seen at coverage level 353, while another at approx. half that coverage indicates that the sampled genome is heterozygous.

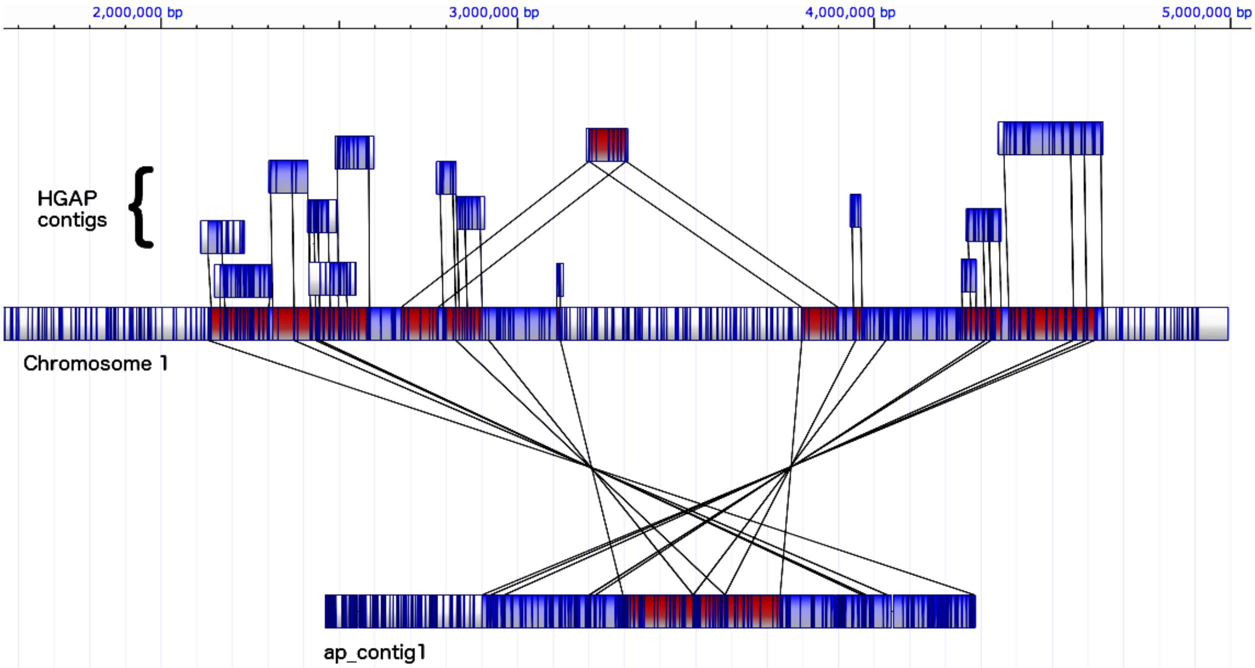

Figure 2. Colourised and annotated screenshot from the OpGen® MapSolver™ software showing a repeat structure in the map Chromosome 1 which allpaths-lg collapsed, but HGAP were not able to fully cover.

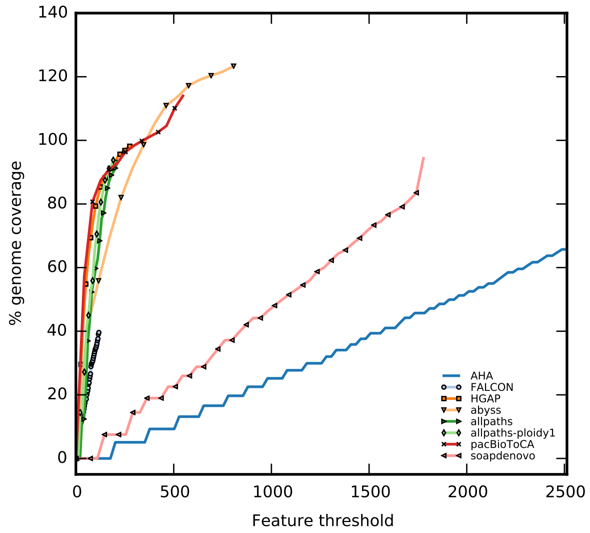

Figure 3. Feature response curve for the feature 'COMPR_MP' in the eight *de novo* assemblies produced. This is a suspected mis-assembly inferred from mapped mate-pair reads that exhibit shorter insert sizes than their expectation. This feature is notable for the AHA and soapdenovo assemblies.

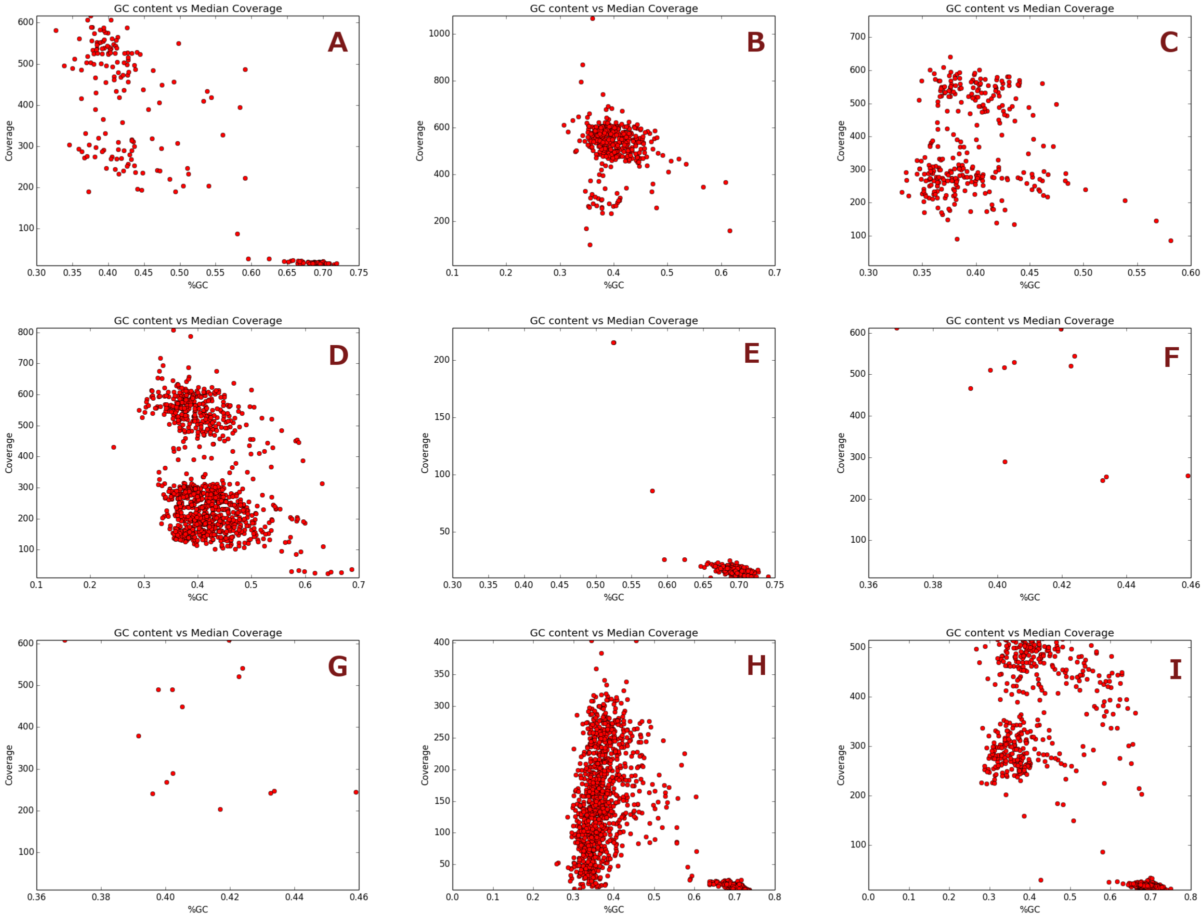

Figure 4. Contig %GC (without outliers) plotted versus median contig coverage (x-axis) for assemblies: (A) AHA, (B) FALCON, (C) HGAP, (D) abyss, (E) allpaths-lg, (F) final assembly chr1-4, (G) assembly chr1-7, (H) pacBioToCA and (I) soapdenovo.

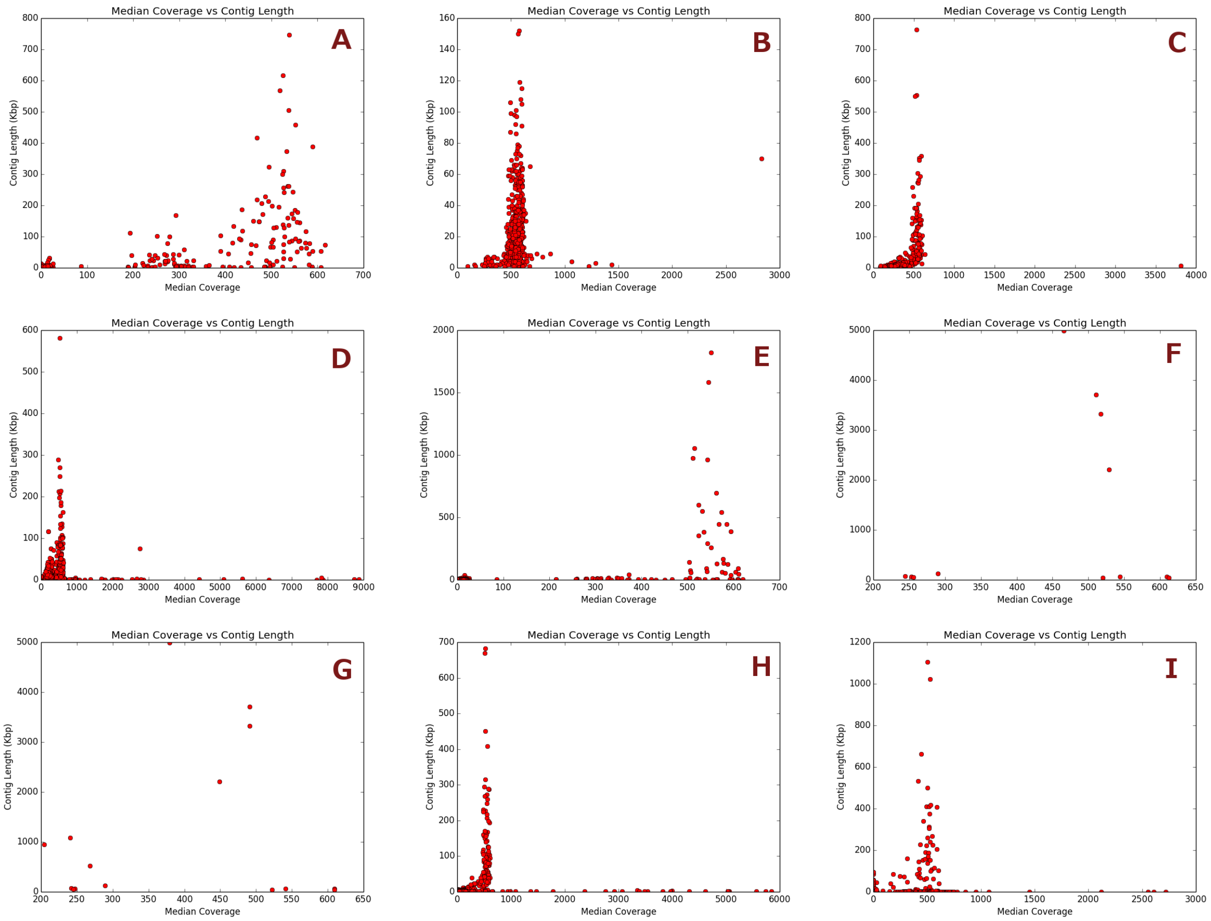

Figure 5. Contig length plotted versus median contig coverage (x-axis) for assemblies: (A) AHA, (B) FALCON, (C) HGAP, (D) abyss, (E) allpaths-lg, (F) final assembly chr1-4, (G) assembly chr1-7, (H) pacBioToCA and (I) soapdenovo.

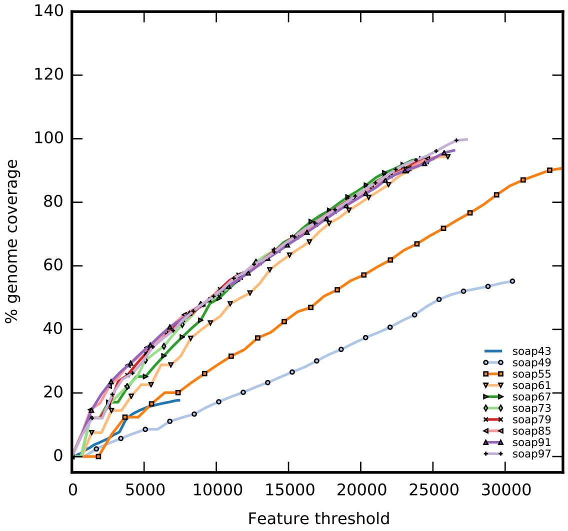

Figure 6. FRC curves for SOAPdenovo assemblies using k-mer size parameters k=(43, 49, .., 97).

Additional tables

Assembler	Complete/Partial	Prots	%Completeness	Total	Average	%Ortho
AHA	Complete	222	89.52	262	1.18	14.86
FALCON	Complete	179	72.18	188	1.05	5.03
HGAP	Complete	245	98.79	280	1.14	11.02
abyss	Complete	244	98.39	406	1.66	32.79
allpaths	Complete	245	98.79	272	1.11	8.57
chr1-7	Complete	238	95.97	322	1.35	30.25
chr1-4	Complete	237	95.56	285	1.2	17.72
pacBioToCA	Complete	245	98.79	312	1.27	17.14
soapdenovo	Complete	165	66.53	179	1.08	5.45
AHA	Partial	239	96.37	294	1.23	17.99
FALCON	Partial	192	77.42	210	1.09	8.33
HGAP	Partial	246	99.19	292	1.19	14.23
abyss	Partial	246	99.19	418	1.7	34.55
allpaths	Partial	246	99.19	285	1.16	12.6
chr1-7	Partial	240	96.77	336	1.4	33.33
chr1-4	Partial	240	96.77	297	1.24	20
pacBioToCA	Partial	246	99.19	336	1.37	22.76
soapdenovo	Partial	217	87.5	255	1.18	12.9

Table 1. Parsed output from CEGMA for all assemblies generated. Of note is the the higher % Orthologous hits when including chromosomes 5 to 7 in the final version of the assembly and the addition of one complete hit.

assembler	n_scaff	n_scaff>1000	N50	N80	max_scf_lgth	Ass_lgth	Ass_lgth_ctgs>1000
soap43	70469	503	206	132	93720	14642557	1706712
soap49	71657	626	748	278	354495	21125096	7076407
soap55	100990	447	165585	7064	1040618	28976782	12392192
soap61	66606	396	263103	49423	1117059	25625475	13038098
soap67	54616	379	244812	41887	1021989	24629116	12923836
soap73	48694	407	158153	16408	1015993	24227966	12714957
soap79	44914	452	106930	14709	1008978	24138274	12827351
soap85	42352	492	77839	12765	1003316	24008233	12772430
soap91	41482	595	61150	10078	999385	24286623	12828418
soap97	33988	454	110017	21640	1002503	23132722	13363549

Table 2. Standard contiguity statistics for test assemblies running SOAPdenovo with a variation of k=(43, 49, .., 97).

Gap type	ALLPATHS+HGAP	Covered by HGAP only	% recovered	% genome recovered
Optical map gaps	1925338	487073	25.30	3.42
Scaffold gaps	333920	224043	67.09	1.57
Ambiguous bases	39426	33130	84.03	0.23
Total	2298684	744246	32.38	5.23

Table 3. A summary of the gaps in the optical map assisted assembly that were covered exclusively by HGAP data. The genome is the length of the optical maps chr1-4: 14230630 bp.

Assembler	Wall time (minutes)	Peak memory usage (Gb)	Data input
AHA	195	3.6	PacBio, PE150, PE650, MPS1/2, MPL1/2
FALCON	2	16.6	PacBio
HGAP	435	1.9	PacBio
abyss	151	12.1	PE150, PE650, MPS1/2, MPL1/2
allpaths	828	103.2	PE150, PE650, MPS1/2, MPL1/2
pacBioToCA	1659	73.9	PacBio, PE150
soapdenovo	43	15.0	PE150, PE650, MPS1/2, MPL1/2

Table 4. The resource usage of the assemblers measured in wall time and peak memory usage. Lastly are the sequencing libraries (see Additional Table 5) used as input. The programs were run on a compute node with 16 Intel Xeon CPU cores and 128 Gb of RAM.

Library	Type	Mean read length	Yield (in Mbp)	Mbp survived trimming
PE150	Paired-end 150bp insert	100	7711	7666
PE650	Paired-end 650bp insert	100	3517	3442
MPS1	Mate-pair short	100	4630	3878
MPS2	Mate-pair short	100	5522	4582
MPL1	Mate-pair long	100	5141	4266
MPL2	Mate-pair long	100	884	808
PacBio	Filtered subreads	1826	730	730

Table 5. A summary of all the sequence libraries showing mean read length (in bp, before trimming) and sequence yield in Mbp before and after trimming. Note that the PacBio data was not trimmed.

References

Blomqvist, Johanna, Thomas Eberhard, Johan Schnürer, and Volkmar Passoth. 2010. "Fermentation characteristics of Dekkera bruxellensis strains." *Applied Microbiology and Biotechnology* 87 (4): 1487–97. doi:10.1007/s00253-010-2619-y.

Borgström, Erik, Sverker Lundin, and Joakim Lundeberg. 2011. "Large scale library generation for high throughput sequencing." *PloS One* 6 (4): e19119. doi:10.1371/journal.pone.0019119.

Carle, G F, and M V Olson. 1985. "An electrophoretic karyotype for yeast." *Proceedings of the National Academy of Sciences of the United States of America* 82 (11): 3756–60. http://www.pubmedcentral.nih.gov/articlerender.fcgi?artid=397866/&tool=pmcentrez/&rendertype=abstract.

Lundin, Sverker, Henrik Stranneheim, Erik Pettersson, Daniel Klevebring, and Joakim Lundeberg. 2010. "Increased throughput by parallelization of library preparation for massive sequencing." *PloS One* 5 (4): e10029. doi:10.1371/journal.pone.0010029.

Schwartz, D C, and C R Cantor. 1984. "Separation of yeast chromosome-sized DNAs by pulsed field gradient gel electrophoresis." *Cell* 37 (1): 67–75. http://www.ncbi.nlm.nih.gov/pubmed/6373014.

Tiukova, Ievgeniia, Thomas Eberhard, and Volkmar Passoth. "Interaction of Lactobacillus vini with the ethanol-producing yeasts Dekkera bruxellensis and Saccharomyces cerevisiae." *Biotechnology and Applied Biochemistry* 61 (1): 40–44. doi:10.1002/bab.1135.
